# Editorial: Improving autism spectrum disorder diagnosis using machine learning techniques

**DOI:** 10.3389/fninf.2024.1529839

**Published:** 2024-12-06

**Authors:** Mahmoud Elbattah, Osman Ali Sadek Ibrahim, Gilles Dequen

**Affiliations:** ^1^Laboratoire MIS, Université de Picardie Jules Verne, Amiens, France; ^2^College of Art, Technology and Environment, University of the West of England, Bristol, United Kingdom; ^3^Department of Computer Science, Minia University, Minya, Egypt

**Keywords:** autism spectrum disorder (ASD), machine learning (ML), artificial intelligence (AI), ASD diagnosis, autism

## 1 Introduction

Autism Spectrum Disorder (ASD) is a complex neurodevelopmental condition characterized by challenges in social communication, repetitive behaviors, and restricted interests (American Psychiatric Association, 2013). Early and accurate diagnosis is critical for effective intervention, enabling individuals with ASD to achieve better developmental outcomes and an improved quality of life. However, traditional diagnostic methods, often reliant on subjective behavioral observations, remain time-intensive and inconsistently accessible. This underscores an urgent need for innovative, scalable, and objective diagnostic tools (Rasul et al., 2024; Jeyarani and Senthilkumar, 2023).

Machine Learning (ML) has emerged as a transformative approach to ASD diagnosis, offering the ability to analyse large, complex datasets and uncover patterns that surpass human capability. For instance, eye-tracking technologies have been extensively utilized to quantify gaze behaviors such as fixations and saccades, as well-established markers of autism. Studies employing Deep Learning have achieved high accuracy in classifying ASD from typically developing individuals based on eye-tracking data (Jeyarani and Senthilkumar, 2023; Alsharif et al., 2024). These technological advancements provide a foundation for developing tools that are not only efficient but also potentially generalizable across diverse populations.

Furthermore, approaches such as transforming gaze scanpaths into visual representations for classification have simplified the diagnostic pipeline, enabling the automation of traditionally laborious processes (Carette et al., 2019). Additionally, unsupervised learning techniques, including clustering of eye-tracking data, have demonstrated potential for extracting unique insights into the variability of ASD presentations (Elbattah et al., 2019). These developments illustrate the growing synergy between AI-driven tools and clinical practices.

Beyond eye tracking, other data modalities such as structural MRI (sMRI), resting-state functional connectivity (rsFC), and multimodal approaches integrating genetic, behavioral, and imaging data have shown promise in identifying robust biomarkers for ASD. These diverse methodologies underscore the importance of leveraging multidimensional data to improve diagnostic precision and reliability (Rasul et al., 2024; Aldrees et al., 2024).

Despite these promising innovations, challenges persist. Standardization of methodologies, reproducibility of results, and the translation of research into clinical applicability remain significant barriers. This Research Topic seeks to address these challenges by presenting cutting-edge research that integrates ML and neuroinformatics to enhance the accuracy, efficiency, and accessibility of ASD diagnostics. By bridging the gap between technology and practice, this collection of studies aims to drive the field toward more effective and equitable solutions for ASD diagnosis.

## 2 Highlights from the contributions in this Research Topic

The articles included in this Research Topic explore various aspects of ASD diagnosis through ML, presenting innovative approaches and significant findings:

### 2.1 A survey of machine learning methods for ASD diagnosis

Eslami et al. provide a comprehensive review of ML models applied to sMRI and fMRI datasets, examining their efficacy in diagnosing ASD and related disorders. The study highlights key advancements in deep learning architectures and identifies limitations such as data heterogeneity and reproducibility challenges.

### 2.2 Novel functional connectivity patterns for ASD identification

Zhao et al. introduce a Boruta-based feature selection technique integrated with Support Vector Machine (SVM) models. Their research uncovers discriminative connectivity patterns within the Default Mode Network (DMN), achieving high classification accuracy and reinforcing the potential of rsFC data in ASD diagnostics.

### 2.3 Bibliometric insights into AI applications in ASD research

Jia et al. conduct a bibliometric analysis, mapping the global research landscape of AI applications in ASD. Their findings highlight trends such as the rise of feature selection and the significance of multimodal integration, providing a roadmap for future studies.

### 2.4 Exploring micro-expressions as ASD biomarkers

Ruan et al. present an exploratory study on using micro-expressions as diagnostic biomarkers. Despite the challenges posed by video data quality, their work emphasizes the need for multimodal approaches combining behavioral and neuroimaging data.

## 3 Broader context and future directions

The contributions in this Research Topic emphasize the multidimensional nature of ASD and the need for a holistic diagnostic framework. Challenges such as the lack of standardization across datasets, ethical considerations in algorithm deployment, and the interpretability of ML models remain relevant. However, the integration of advanced computational methods with clinical expertise opens avenues for personalized treatment strategies and early intervention protocols.

We envision that future research should focus on:

- **Data diversity and multimodal integration:** combining imaging, genetic, and behavioral data to enhance model robustness.
- **Interpretable AI:** Developing transparent algorithms that clinicians can trust and use effectively.
- **Generative AI for personalized diagnostics:** leveraging the recent advancements of Generative AI, such as large language models (LLMs) and multimodal generative systems, to create tools that can synthesize patient-specific insights by integrating textual, visual, and clinical data. These models could enable personalized ASD assessments, simulate diagnostic scenarios, and even provide tailored recommendations for clinicians.
- **Global collaboration:** standardizing datasets and fostering cross-disciplinary partnerships to address disparities in access and implementation.
